# The Association Between Brominated Flame Retardants Exposure and Liver-Related Biomarkers in US Adults

**DOI:** 10.3390/toxics12120852

**Published:** 2024-11-26

**Authors:** Yuqing Chen, Yulan Cheng, Jialing Ruan, Donglei Huang, Jing Xiao, Xinyuan Zhao, Jinlong Li, Jianhua Qu, Xiaoke Wang

**Affiliations:** 1 Nantong Key Laboratory of Environmental Toxicology, Department of Occupational Medicine and Environmental Toxicology, School of Public Health, Nantong University, Nantong 226019, China; 2317320002@stmail.ntu.edu.cn (Y.C.); nanyu295@163.com (Y.C.); ruanjialing0917@163.com (J.R.); huangdlntly@163.com (D.H.); xiaoj_1980@ntu.edu.cn (J.X.); zhaoxinyuan@ntu.edu.cn (X.Z.); 2 School of Pharmacy, Nantong University, Nantong 226001, China; jinlongli@ntu.edu.cn

**Keywords:** NHANES, brominated flame retardants, liver-related biomarkers, liver function tests, BKMR

## Abstract

**Background:** Emerging studies demonstrate that exposure to brominated flame retardants (BFRs) can have harmful effects on human health. Our study focused on the relationship between exposure to various BFRs and markers of liver function. **Methods:** To further explore the association between BFR exposure and liver function impairment, we used data from the National Health and Nutrition Examination Surveys (NHANES) for three cycles from 2009 to 2014, leaving 4206 participants (≥20 years of age) after screening. Nine BFRs and eight liver function tests (LFTs) were measured in the participants’ serum to represent BFRs and liver function impairment in vivo. To investigate whether there is a relationship between BFRs and health outcome, statistical research methods such as the weighted linear regression model, restricted cubic spline (RCS), weighted quantile sum (WQS), quantile-based g computing (QGC), and the Bayesian Kernel Machine Regression (BKMR) were used to evaluate the correlation between serum BFRs and LFTs. **Results:** The studies reveals that exposure to BFRs is associated with liver function biomarkers. In a weighted linear regression model, we found that PBB153, PBDE99, PBDE154, PBDE209, PBDE85 exposure was positively correlated with AST, ALT, GGT, ALP, TP, and SL risk. In RCS model, the nonlinear relationships between PBB153 and AST, ALT, and GGT and PBDE209 and ALT and TP are the most significant. The exposure to combined BFRs was positively correlated with AST, ALT, and GGT in WQS and QGC models. BKMR analysis showed that BFR exposure was positively correlated with AST, ALT, ALP, and GGT. **Conclusions:** Exposure to BFRs is associated with liver function impairment, suggesting that BFR exposure is potentially toxic to the human liver, but more in-depth studies are needed to explore this correlation.

## 1. Introduction

When a fire rages into a catastrophe and the flames relentlessly consume everything, it can cause serious damage. So, to prevent fires, scientists have creatively added some flame-retardant substances to flammable items to effectively reduce the risk of fire, and these flame-retardant additives are called flame retardants (FRs). Brominated flame retardants (BFRs), as an important subcategory, work by trapping free radicals in the gas phase with bromine, thereby reducing the rate of ignition and combustion [1]. BFRs are widely used in products such as plastics and electronic equipment, as well as in the construction and textile industries [2]. The most common types of BFRs include polybrominated diphenyl ethers (PBDEs) and polybrominated biphenyls (PBBs) [3]. Of these, PBDEs, because of their low price and high flame retardant effect, have an estimated historical production of 1,900,000 tons globally [4]. Consequently, the extensive use of BFRs led to their ubiquitous presence in the environment. BFRs have even been detected in the Antarctic and Arctic regions, which demonstrates the ability of them to migrate over long distances [5]. To protect human health and environmental safety, restrictions on the production and use of BFRs have now been put in place. For example, the United Nations Environment Program has listed some BFRs such as PBDEs and PBBs as persistent organic pollutants (POPs) under the Stockholm Convention. The governments of some countries have also successively implemented many actions and promulgate laws to restrict the use of BFRs [6,7]. 

Though production has been forbidden, BFRs will remain in the environment for a long time because of their persistence, non-degradability, and long half-life [8]. The estimated intake of BFRs occurs mainly through inhalation of indoor air and dust, dietary intake, and dermal contact [9]. Previous studies have discovered that BFRs are present in human blood serum, fat, liver tissues, placenta, umbilical cord serum, and breast milk, among other substances [1]. Due to their lipophilic nature, BFRs possess a propensity to amass in organs like the liver, which is abundant in lipids [8]. According to a recent study, BFRs can cause lipid metabolism disorders, having a great impact on the accumulation of lipids in hepatocytes, and they may increase the risk of metabolic dysfunction in adipose tissue, thereby affecting liver metabolism [10]. Meanwhile, PBBs and PBDEs in rats caused an increase in hepatic phospholipids, inducing hepatocellular swelling and necrosis, hepatic tumor nodules, hepatocellular adenomas, and carcinomas [11]. Zeng et al. indicated that the disruption of normal liver metabolism may occur via the induction of oxidative stress and inflammatory responses, thereby resulting in hepato-toxic effects and causing liver injury [12,13].

As an important target organ of most POPs, the liver is a highly active metabolic organ in the body, involved in the regulation of blood volume, storage of gluconeogenesis and glycogen, cholesterol metabolism and bile acid synthesis, and exogenous metabolism and detoxification [14,15,16]. Since their development in the 1950s, liver function tests (LFTs) have been commonly used to assess liver function, to identify areas of hepatic injury, and to aid in the differential diagnosis [17]. Common biomarkers of liver function typically include alanine aminotransferase (ALT) and aspartate aminotransferase (AST), alkaline phosphatase (ALP), gamma-glutamyltransferase (GGT), and serum bilirubin [17].

In the current research, we utilized data from the National Health and Nutrition Examination Study (NHANES) and different statistical analysis models to explore the relationship between BFRs and liver function impairment and to investigate potential confounding and effects [18].

## 2. Materials and Methods

### 2.1. Study Population

NHANES is a study conducted by the National Center for Health Statistics (NCHS) to evaluate the health and nutrition status of adults and children in the United States. NHANES began in the 1960s and was restructured into a continuous research program in 1999. It is an innovative combination of interviews and medical examinations, in which participants are interviewed by trained professionals with questionnaires about basic demographic characteristics, socio-economic, dietary, and health-related issues. Specific provisions of the inspections department include medical, dental, and physiological measurement, as well as laboratory tests, etc. [19]. 

**Figure 1:**
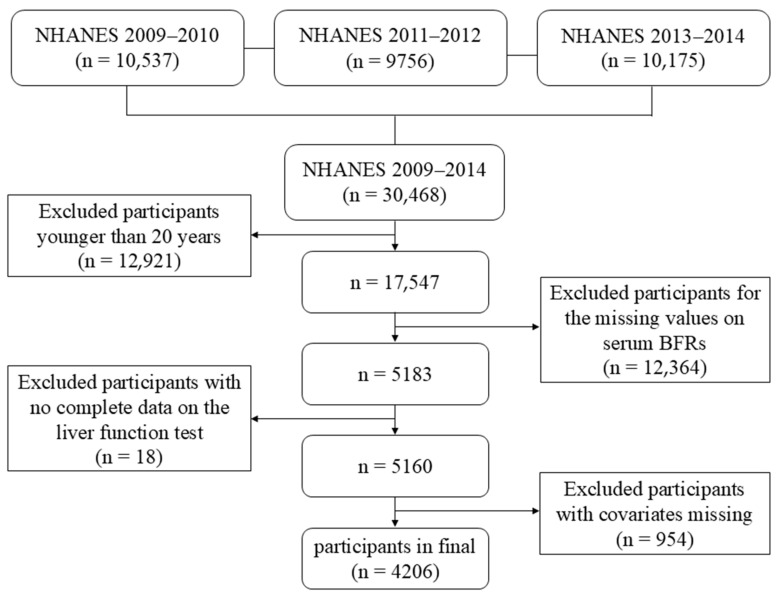
Flow diagram for selecting eligible participants from NHANES.

This study used data from a total of 30,468 participants in NHANES from 2009 to 2014. Subjects with the following relevant characteristics were excluded: (1) age < 20 years (n = 12,921); (2) serum BFR data missing (n = 12,364); (3) lack of liver function test data (n = 18); (4) no information on relevant covariates (n = 954), which include gender, race, education level, income, marital status, BMI, serum cotinine level, alcohol consumption, hypertension, diabetes, etc. The final analysis included 4206 participants. The participant selection process was shown in Figure 1.

### 2.2. Exposure Variables

A total of 12 BFRs were detected in participants in the NHANES database. Serum BFR concentrations were checked using automatic liquid–liquid extraction and subsequent sample cleaning. On the NHANES web pages, the detection method includes a detailed description of BFR [20]. In order to ensure the strength of the results and the representativeness of the data, referring to some previous studies of BFRs in NHANES [3,21], only 9 BFRs with a detection rate of more than 75% in the database were included in the 3 cycles of this study, namely PBB153 (code: LBCBB1), 2,4,4′-Tribromodiphenyl ether (PBDE28) (code: LBCBR2), 2,2′,4,4′-Tetrabromodiphenyl ether (PBDE47) (code: LBCBR3), 2,2′,3,4,4′-Tetrabromodiphenyl ether (PBDE85) (code: LBCBR4), 2,2′,4,4′,5-Pentabromodiphenyl ether (PBDE99) (code: LBCBR5), 2,2′,4,4′,6-Pentabromodiphenyl ether (PBDE100) (code: LBCBR6), 2,2′,4,4′,5,5′-Hexabromodiphenyl ether (PBDE153) (code: LBCBR7), 2,2′,4,4′,5,6′-Hexabromodiphenyl ether (PBDE154) (code: LBCBR8), and Decabromodiphenyl ether (PBDE209) (code: LBCBR11). Serum BFR concentrations below the lower limit of detection (LLOD) were estimated by dividing the LLOD value by the square root of 2. The specific contents can be seen in Table S1.

### 2.3. The Indicators of LFTs

During the study period, we selected a total of 8 indicators to detect liver function, namely ALT (code: LBXSATSI), ALB (code: LBDSALSI), AST (code: LBXSASSI), TBIL (code: LBDSTBSI), GGT (code: LBXSGTSI), ALP (code: LBXSAPSI), TP (code: LBDSTPSI), and AST/ALT ratio were used. The indicators used in this article are widely recognized. Previous studies have confirmed their indicative and scientific nature in reflecting human liver function [22,23,24]. ALT and AST are transaminases, which increase rapidly during acute liver injury, indicating that the liver cell membrane may be damaged, or liver cell necrosis [25]. ALB and TP indicate the synthesis capacity of the liver, and a decrease in these two indicators indicates that liver synthesis is limited [25]. Serum TBIL concentration is regulated by both the bilirubin production rate and hepatocyte clearance rate [26]. GGT is often used clinically to determine the cause of cholestasis. One of the indicators of cholestasis is an elevated ALP. AST/ALT refers to the ratio of AST and ALT concentrations. The detection method was Beckman UniCel DxC800 Synchron. In NHANES, the processing and analysis of the specific instructions are included in the laboratory method [27]. Different methods were used to detect each index of the serum LFTs. The activity of ALT, AST, GGT, and ALP can be determined by the kinetic rate method. ALB was quantitatively determined by two-color digital end point method. TP was determined by using the timed-rate biurea method [28].

### 2.4. The Fibrosis-4 Index (FIB-4)

To further investigate whether BFR exposure caused more severe damage to liver function, FIB-4 was used to assess liver fibrosis [29,30]. FIB-4 was calculated using the formula (age × AST)/(platelet count × ALT1/2). Higher FIB-4 values indicate more severe liver fibrosis.

### 2.5. Covariates

All covariates included in this study were selected based on previous studies on BFRs and liver function impairment [21,28]. Among the included covariates, the age of the participants was analyzed as a continuous variable. Other covariables were analyzed as categorical variables: gender (male, female), race (Mexican American, other Hispanic, non-Hispanic white, non-Hispanic black, etc.), education level (below 9th grade, 9–11 grades, high school diploma or above), marital status (married/partnered, widowed/divorced/separated/unmarried), household income to poverty ratio (≤1.30, 1.31–3) 0.50, >3.50), body mass index (<25 kg/m^2^, 25–30 kg/m^2^, ≥30 kg/m^2^), alcohol consumption, high blood pressure, diabetes, and vigorous or moderate physical activity in the past seven days. Serum cotinine concentrations were used to reflect smoking status.

### 2.6. Statistical Analyses

In this study, all analyses used R version 4.2.3, and a bilateral *p*-value of 0.05 was used to pick out statistical significance. In the descriptive analysis, the median and quartile distance (IQR) or geometric mean and standard deviation of the continuous variables were calculated for 9 BFRs, 8 LFTs, and FIB-4. After the normality test, it was found that the BFRs, LFTs, and FIB-4 are skewed. Therefore, natural logarithm (ln) transformation was first performed on the data to transform it into an approximate normal distribution. Spearman’s correlation coefficient was used to evaluate the pairwise correlation between ln-transformed BFRs.

Since NHANES uses a complex multi-stage sampling design, we use a weighted model in the multiple regression analysis. We first included individual BFR concentrations as continuous variables in regression models and evaluated their respective linear associations with LFTs and FIB-4. Secondly, the continuous variable BFR model was adjusted to order variables, a linear trend test was performed, and OR and 95% confidence interval were calculated. By using quartiles, the BFR concentrations were divided into four equal fractions, with the first quartile as the control, and the other quartiles defined as Q2, Q3, and Q4, respectively. We adjusted the model for all potential confounding factors. Finally, we further fitted the data with restricted cubic splines (RCS) to analyze the nonlinear relationship between BFRs and LFTs. A total of 4 nodes are set in the model to maintain smoothness and stability while avoiding the precision decline caused by over-fitting.

In addition, in order to deal with problems such as high data dimension and multicollinearity when BFRs are exposed as a combination of mixed chemicals, weighted quantile and regression (WQS) analysis were employed in this study. By utilizing the interquartile interval of serum BFR concentration and weighting, the data were divided into training sets and validation sets, the stable weights were calculated, and the effects of multiple factors were combined with an index for regression analysis, aiming to evaluate the overall effect of BFR exposure and find out which BFRs produce toxic effects after exposure. However, due to the unidirectional nature of WQS, it solely detects mixed effects that are positively or negatively correlated with a given result. There was no previous evidence on the effect directions of the nine BFRs and eight LFTs, so two runs were needed to test the association between the positive and negative directions. To more accurately assess the effects of mixed BFR exposure and overcome the limitations of the WQS model in the relational direction, we also employed a quantile-based g computation (QGC) method to analyze the QGC model through “qgcomp” in the R package.

Considering this analysis, the damage effect of mixed BFR exposure on human liver function should be considered from the perspective of overall synthesis, so the Bayesian Kernel Machine Regression (BKMR) was also utilized in this study. BKMR is a Bayesian non-parametric regression method that can consider nonlinearity and non-additive relations at the same time. The core idea is to transform the regression problem into an inner product calculation in the feature space, and to measure the similarity between samples through the kernel function. Using the “bkmr” package in R software (version 3.6), the combined effects of BFRs exposure on liver function and the potential interactions between each BFR were evaluated.

## 3. Results

### 3.1. Basic Characteristics of Participants

Table 1 revealed the basic demographic characteristics of the participants included in this research in the NHANES database from 2009 to 2014. The 4206 participants were 49.16 (17.73) years old, with slightly fewer males than females (49.2% vs. 50.8%), predominantly non-Hispanic white (44.3%), well educated (54.3%), married or in a relationship (59.1%), and hypertensive (63.6%). Many reported more than moderate exercise in the past seven days (88.2%). Table 2 describes the concentration distribution of 9 serum BFRs and 8 serum liver function indicators in the human body. We calculated geometric means and 95% confidence intervals for each BFR and liver function measure, as well as quartiles. The serum concentration of PBDE47 was significantly higher than that of other BFRs. Table S1 shows the detection rate and distribution of 9 BFRs in the three cycles analyzed.

### 3.2. Association Between Each Kind of Serum BFR with LFTs

The nine BFRs and eight LFTs detected in this study were all continuous variables. In Figure S1, all variables were analyzed using Pearson’s correlation coefficients. Spearman’s correlation analysis showed low to high correlation among the nine BFRs (r range: 0.24~0.94), among which PBDE47, PBDE88, and PBDE99 had the highest correlations (r = 0.94, heat map). However, correlations between covariates and effects were low. This also indicates that there may be multicollinearity in the data analyzed by the general regression method, so other models were used for further analysis. 

**Figure 2:**
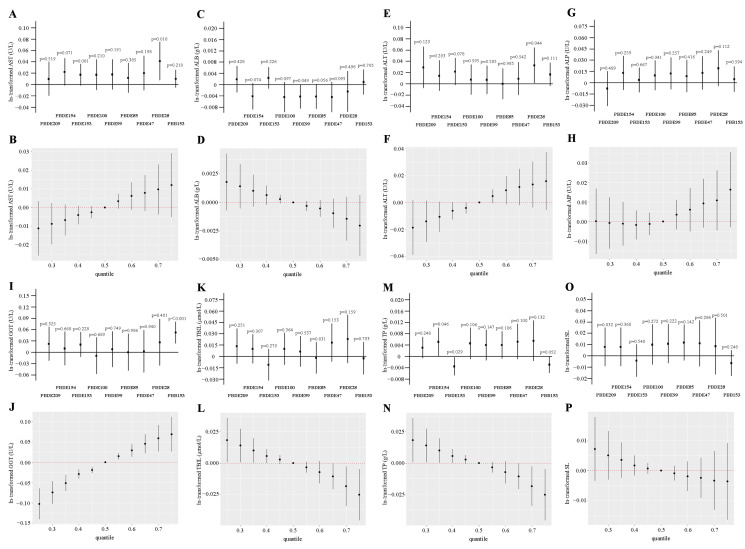
Associations between ln-transformed serum BFRs levels and AST, ALB, ALT, ALP, GGT, TBIL, TP, and SL levels based on survey-weighted regression, and joint effects of the BFR mixture on the liver function test levels estimated by Bayesian Kernel Machine Regression (BKMR) (N = 4206). All of the models are adjusted to demographic characteristics (gender, age, race, educational levels, marital status and PIR), lifestyle (BMI categories, cotinine levels and alcohol consumption) and self-reported of hypertension, diabetes conditions, and activity. The estimated β and 95% confidence intervals for associations between single phthalate and (**A**) AST, (**C**) ALB, (**E**) ALT, (**G**) ALP, (**I**) GGT, (**K**) TBIL, (**M**) TP, and (**O**) SL. The overall effect of the phthalate mixture on (**B**) AST, (**D**) ALB, (**F**) ALT, (**H**) ALP, (**J**) GGT, (**L**) TBIL, (**N**) TP, and (**P**) SL levels.

Multiple logistic regression analysis was employed in our study to evaluate the independent influence of exposure on the outcome. Based on linear regression analysis, after adjusting for a series of confounding variables (including race, education level, marital status, BMI, cotinine level, etc.), the relationship between BFRs and liver function impairment markers in participants was clearly revealed, as shown in Figure 2. The first percentile was set as the reference quartile, and the second, third, and fourth quartiles were compared with the reference quartile. In particular, all three quartiles demonstrated an elevated risk of AST, ALT, and GGT following PBB153 exposure. The level of ALP increased after PBB153 exposure at the second percentile comparison. When Q3 was compared with Q1, it was found that exposure to PBDE99, PBDE154, and PBDE209 was positively correlated with ALT, PBDE99, PBDE154, and GGT, and PBDE209 and TP. Compared with Q2, Q3, and Q1, the level of TP increased after PBDE154 exposure. Comparing Q2 with Q4 and Q1, PBDE85 was associated with an increased level in SL. In addition, ALB decreased after PBDE47 exposure when Q3 was compared with Q1. In the Q4 comparison, exposure to PBDE99, PBDE100, and PBDE154 was negatively correlated with ALB. Comparing Q2 to Q4 and Q1, PBB153 was associated with a reduced level of SL. In addition, Tables S2 and S3 show the relationship between each BFR and the subjects’ liver function markers.

**Figure 3:**
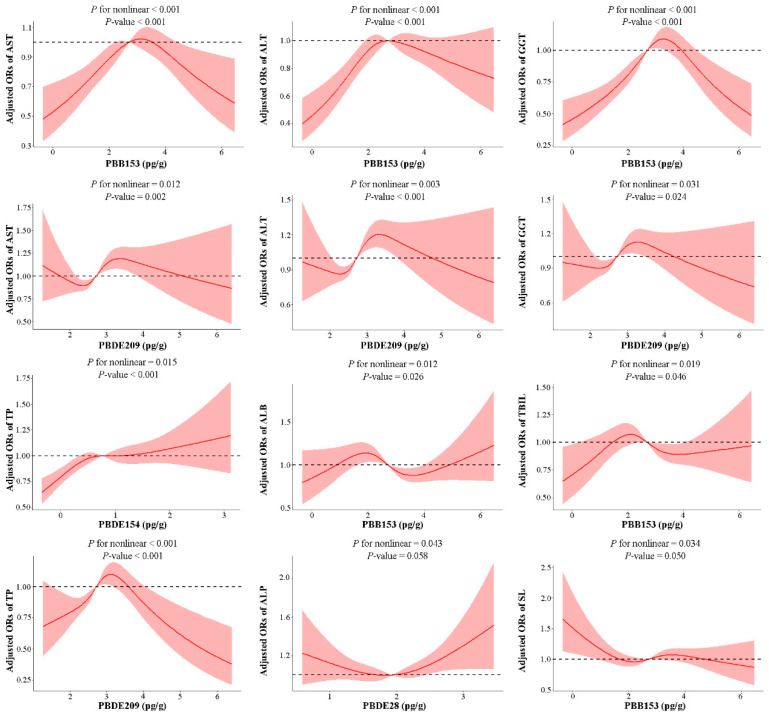
The significant continuous relationship of ln-transformed serum BFR levels associated with depression risk based on RCS analysis. The red solid lines represent the ORs, and the red shadow range represents the 95% CIs. The horizontal dashed line represents the reference odds ratio of 1.0. All of the models are adjusted for demographic characteristics (gender, age, race, educational levels, marital status, and PIR), lifestyle (BMI categories, cotinine levels and alcohol consumption), and self-reported hypertension, diabetes conditions, and activity.

The RCS model was utilized to analyze the nonlinear relationship between BFRs and LFTs, and the meaningful part is shown in Figure 3. There are nonlinear correlations between PBB153 in BFRs and AST, ALB, ALT, GGT, TBIL, and SL; between PBDE28 and ALP; between PBDE154 and TP; and between PBDE209 and AST, ALT, GGT, and TP in LFTs. The nonlinear relationship between PBB153 and AST, ALT, and GGT, and between PBDE209 and ALT and TP was significant (*p* < 0.001). In addition, the RCS models of all the BFRs and LFTs are shown in Figures S2–S5.

### 3.3. WQS and QGC Models to Assess the Associations Between Co-Exposure of BFRs and LFTs

**Figure 4:**
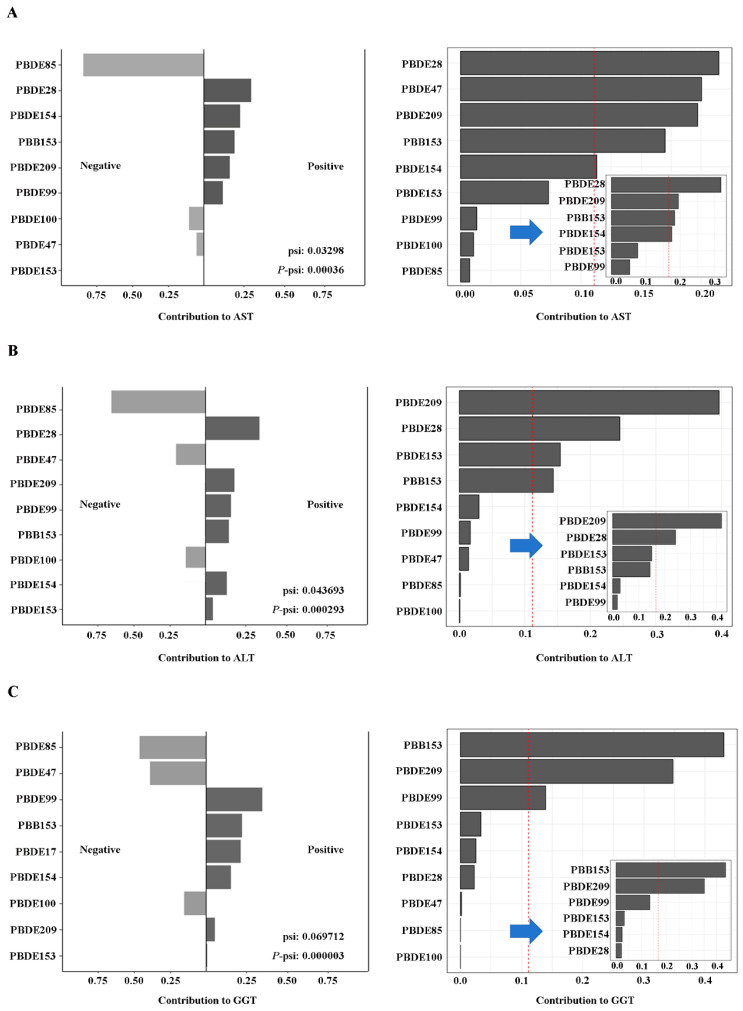
Association between ln-transformed serum BFRs mixture and the risk of liver function impairment in all participants, as assessed via quantile-based g-computation (first step) and weighted quantile sum regression (second step after exclusion of exposure factors with negative weights). (**A**) The risk of AST in all participants; (**B**) the risk of ALT in all participants; (**C**) the risk of GGT in all participants. All of the models are adjusted for demographic characteristics (gender, age, race, educational levels, marital status, and PIR), lifestyle (BMI categories, cotinine levels, and alcohol consumption), and self-reported of hypertension, diabetes conditions, and activity. The arrows in the figure indicate that all the positive correlation of the single BFR obtained in the QGC model is incorporated into the WQS model, and the positive analysis is performed again.

Association between ln-transformed serum BFRs mixture and the risk of liver function impairment in all participants, as assessed via quantile-based g-computation (first step) and weighted quantile sum regression (second step after exclusion of exposure factors with negative weights). As shown in Figure 4 and Table S4, exposure to BFRs and AST (OR: 1.015; 95% CI: 0.994, 1.035), ALT (OR: 1.049; 95% CI: 1.020, 1.078), and GGT (OR: 1.055; 95% CI: 1.013, 1.099) was positively correlated. In this study, PBDE28, PBDE209, PBB153, PBDE154, PBDE153, and PBDE99 were the main factors that increased the level of AST. PBDE209, PBDE28, PBDE153, PBB153, PBDE154, and PBDE99 were the major contributors to the elevated risk of ALT. PBB153, PBDE209, PBDE99, PBDE153, PBDE154, and PBDE28 were the major contributors to the increased risk of GGT. Consistent conclusions about potential risk factors were obtained from QGC and WQS.

### 3.4. BKMR Model to Assess the Associations Between Co-Exposure of BFRs and LFTs

The association of BFRs with AST, ALB, ALT, ALP, GGT, TBIL, TP, and SL levels in the individuals was investigated with weighted regression, while the combined effect of BFRs on LFTs was estimated by BKMR (N = 4206). All models in Figure 2 were adjusted for confounding variables. PBDE28 was positively correlated with AST (*p* = 0.018) and ALT (*p* = 0.044), PBB153 was significantly correlated with GGT (*p* < 0.001), and PBDE154 was positively dose-dependent with TP (*p* = 0.046). In Figure 2, the BKMR analysis also showed a positive dose–response relationship between BFRs and AST, ALT, ALP, or GGT. When all BFR concentrations were at or above the 55th and 60th percentiles of the median level, respectively, AST, ALT, ALP, and GGT increased significantly compared to in the 50th percentile. When BFR concentrations were all above the 55th percentile compared to the median, a significant overall negative effect of BFR concentration on ALB was observed. When BFR concentration levels were at or below the 45th percentile, the overall effect of the mixture on TBIL, TP, and SL was significant compared to the 50th percentile, suggesting that TBIL, TP, and SL may decline with increasing BFRs.

### 3.5. Association Between Each Kind of Serum BFR with Liver Fibrosis Indicator

Based on screening of the remaining 4206 participants in Figure 1, we calculated the FIB-4 index for each individual according to the formula, and eight individuals were excluded due to incomplete data. Among the remaining 4198 participants, weighted linear regression was used to analyze the association between BFRs exposure and FIB-4. As shown in Table S5, compared Q3 with Q1, FIB-4 increased after PBB153 exposure. When Q2 and Q3 were compared with Q1, it was found that the exposure of PBDE47 was positively correlated with FIB-4. In Figure S6, further QGC and WQS analyses revealed that PBB153 was the main factor that increased the level of FIB-4.

## 4. Discussion

Using NHANES data from 2009 to 2014, the separate and combined effects between BFRs and liver function impairment indicators were analyzed, considering data independence and nonlinearity, and adjusting for confounding variables. In the analysis of the weighted linear regression model, we found that PBB153, PBDE99, PBDE154, PBDE209, and PBDE85 exposure was positively correlated with AST, ALT, GGT, ALP, TP, and SL risk. In the RCS model, the nonlinear relationships between PBB153 and AST, ALT, and GGT, and between PBDE209 and ALT and TP were the most significant. Through WQS and QGC model analysis, common exposure to BFRs was positively correlated with AST, ALT, and GGT. Consistent results were obtained in the BKMR analysis, with positive dose relationships between BFRs exposure and AST, ALT, ALP, and GGT.

In a recent statistical study of BFRs and liver function alongside non-alcoholic fatty liver disease in the US population, serum BFR concentration was also found to be positively correlated with liver function. The study analyzed data from 2005 to 2016 from 3108 respondents in NHANES [31]. Our study used data from 2009 to 2014 on 4206 NHANES participants. In addition to using statistical methods such as linear regression analysis, RCS, WQS, and QGC, BKMR was also used in our study to analyze the effects of combined multiple BFR exposure on LFTs, and more robust conclusions were obtained. Our study uses a broader variety of statistical methods and a larger sample size of data, making it more reliable and enabling a more in-depth analysis of the association between BFRs and liver function markers.

Although BFRs have been banned for many years, most BFRs can still be detected in the serum of the adult population in America [21]. BFRs have been widely used as a class of flame-retardant chemicals. Whether they can cause liver function change has been discussed in animal experiments and epidemiological studies. Until now, BFRs were commonly found in the livers of cormorants, herrings, and penguins [32,33]. Tested tilapia models exposed to BFRs showed that BFRs affected the activity of biomarkers in the fish liver at very low concentrations [34]. Another experiment also found that rat hepatocytes were highly sensitive to PBB, and a higher HBB threshold was found in human hepatocytes than in rat hepatocytes [35]. Studies and analyses have found that BFRs may cause oxidative damage and apoptosis, resulting in liver function biomarker changes, through influencing mechanisms such as glucose and lipid metabolism disorders and mitochondrial damage [36]. In our study, the population data in NHANES were used explore the relationship between BFR exposure and liver function markers, and a conclusion similar to the above experimental study was obtained: there is a positive correlation between BFR exposure and liver function detection indicators.

Therefore, the related mechanisms also need to be explored. BFRs are lipophilic and therefore accumulate in the liver. More and more studies have found that BFR may interfere with liver glucose and lipid metabolism. There have been reports of glucose and lipid metabolism disorders in mouse BFR exposure models, and of potential interference with adipokine expression and insulin secretion through influence on the expression of PPARγ and AMPKα, which play a crucial role in glucose and lipid metabolism, leading to glucose and lipid metabolism disorders [37]. Another study found that when PPARα /γ receptor agonists were used to treat NAFLD and diabetic dyslipidemia, ALT and AST indexes were significantly reduced [38]. Some studies have suggested that BFR may affect mitochondrial function, and the serum tests of occupationally exposed people in BFR manufacturing plants have revealed that their mitochondrial DNA copy number is reduced [39]. Mitochondrial dysfunction plays an important role in liver injury [40]. It has also been found that BFR can cause oxidative stress in the liver, and that GSH concentrations in the liver of Zebrafish gradually decreased after exposure to BFR, showing a significant dose–effect relationship [41]. Borax has been reported to be beneficial to cellular oxidative stress, and one study used borax to ameliorate liver injury by improving tissue REDOX homeostasis [42].

Our research has several advantages. First, the data in our research came from the NHANES database, which is a large sample database with national representation that adopts a sophisticated stratified and multi-stage probability sampling method, improving the universality and validity of our research results. In addition, we used multiple logistic regression, RCS analysis, a WQS model, QGC analysis, and the BKMR model to drastically investigate the independent and general effects of BFR exposure on liver function impairment markers. BKMR is a Bayesian non-parametric regression method that can more flexibly solve the high collinearity problem and the interaction of exposure variables when BFRs are jointly exposed.

## 5. Limitations

However, some limitations to our study must be recognized. First, because this is a cross-sectional study, we cannot deduce a causal relationship between BFR exposure and liver function impairment markers. Second, although this study used linear logistic regression to adjust for confounding variables such as age, sex, ethnicity, and education level, potential confounding variables may still exist. Third, although there is a positive correlation between BFR exposure and liver function impairment markers, the mechanism still needs further exploration. Fourth, the information used in this research available in the NHANES database only reports up to 2014, and the latest data from recent years are not available. Fifthly, based on the prescription drug information provided on the NHANES official website, it is difficult to clearly and comprehensively determine which drugs will change the blood lipid and blood glucose levels of the population. Sixth, as POPs, BFRs degrade slowly in the body, and it is difficult to determine the exposure time of different populations, so there is a bias in the exposure time, which may have some influence on the study results. In addition, because the LFTs we selected are indicators in blood tests, the serum level reflects the long-term and chronic exposure of the human body. However, some of these LFTs, such as ALT and AST, are transaminases that increase rapidly during acute liver injury. Therefore, bias may also occur in the detection of effects. Therefore, more longitudinal studies are required to determine the causal relationship between long-term exposure to BFR and liver function impairment.

## 6. Conclusions

In this research, the individual and general effects of BFR exposure were significantly associated with eight LFTs in U.S. adults, suggesting that BFR exposure may be associated with liver function impairment markers. We found that AST, ALT, and GGT were most significantly affected by brominated flame retardants, and PBB153, PBDE28, and PBDE209 exhibited the highest importance. This suggests the need to continue searching for safer alternatives to BFR for human health. In the future, it is suggested that further prospective cohort studies are required to continuously monitor the damage caused by BFRs to liver function and to explore the causal relationship and exact mechanisms between them.

## Figures and Tables

**Table 1:** Basic demographics of the study sample participating in NHANES 2009–2014 (N = 4206).

Characteristics	Overall
**No. subjects**	4206
**Age (%) ^a^**	49.16 ± 17.73
20–40 years	1440 (34.2)
40–60 years	1378 (32.8)
≥60 years	1388 (33.0)
**Sex (%) ^b^**	
Male	2071 (49.2)
Female	2135 (50.8)
**Race/ethnicity (%) ^b^**	
Mexican American	575 (13.7)
Other Hispanic	418 (9.9)
Non-Hispanic White	1864 (44.3)
Non-Hispanic Black	877 (20.9)
Other Race—including multi-racial	472 (11.2)
**Educational level (%) ^b^**	
Below High School	971 (23.1)
High School	952 (22.6)
Above High school	2283 (54.3)
**Marital status (%) ^b^**	
Married/living with partner	2486 (59.1)
Widowed/divorced/separated/never married	1720 (40.9)
**Poverty income ratio (%) ^b^**	
≤1.3	1388 (33.0)
1.3–3.5	1525 (36.3)
>3.5	1293 (30.7)
**Body mass index (%) ^b^**	
<25 kg/m^2^	1223 (29.1)
25–30 kg/m^2^	1398 (33.2)
≥30 kg/m^2^	1585 (37.7)
**Cotinine level (%) ^b^**	
Below LLOD	1194 (28.4)
Above LLOD	3012 (71.6)
**Alcohol consumption (%) ^b^**	1124 (26.7)
**Hypertension (%) ^b^**	2673 (63.6)
**Diabetes (%) ^b^**	542 (12.9)
**Strenuous/moderate activity in the past seven days (%) ^b^**	3709 (88.2)

^a^ Mean value and standard deviation (SD). ^b^ Number of participants and percentage.

**Table 2:** Geometric means and quartiles of serum BFRs, liver function tests, and fibrosis-4 index, NHANES 2009–2014 (N = 4206).

	GM (95% CI) ^a^	Median (IQR) ^b^
**Serum BFRs (pg/g)**		
PBB153	14.274 (13.805, 14.761)	14.980 (6.627, 27.130)
PBDE28	6.919 (6.807, 7.036)	6.992 (4.739, 10.010)
PBDE47	122.688 (120.422, 124.961)	118.700 (81.780, 183.600)
PBDE85	2.344 (2.293, 2.394)	2.210 (1.485, 3.710)
PBDE99	23.655 (23.150, 24.167)	22.280 (14.600, 37.300)
PBDE100	25.004 (24.533, 25.483)	23.830 (16.067, 37.070)
PBDE153	54.423 (53.303, 55.590)	51.030 (33.930, 82.690)
PBDE154	2.222 (2.177, 2.268)	2.150 (1.419, 3.414)
PBDE209	15.881 (15.611, 16.167)	15.200 (10.900, 20.960)
**Liver function tests**		
AST (U/L)	24.144 (23.903, 24.410)	23.000 (20.000, 28.000)
ALB (g/L)	42.403 (42.309, 42.521)	43.000 (40.000, 45.000)
ALT (U/L)	22.293 (21.977, 22.624)	21.000 (16.000, 28.000)
ALP (U/L)	64.563 (63.944, 65.170)	64.000 (53.000, 79.000)
GGT (U/L)	21.458 (21.031, 21.889)	19.000 (14.000, 30.000)
TBIL (μmol/L)	11.126 (10.990, 11.257)	11.970 (8.550, 13.680)
TP (g/L)	71.285 (71.165, 71.450)	71.000 (68.000, 74.000)
AST/ALT	1.083 (1.074, 1.093)	1.092 (0.903, 1.313)
**Fibrosis-4 index**	0.364 (0.358, 0.370)	0.369 (0.248, 0.530)

PBB153: 2,2′,4,4′,5,5′-Hexabromobiphenyl; PBDE28: 2,4,4′-Tribromodiphenyl ether; PBDE47: 2,2′,4,4′-Tetrabromodiphenyl ether; PBDE85: 2,2′,3,4,4′-Tentabromodiphenyl ether; PBDE99: 2,2′,4,4′,5-Pentabromodiphenyl ether; PBDE100: 2,2′,4,4′,6-Pentabromodiphenyl ether; PBDE153: 2,2′,4,4′,5,5′-Hexabromodiphenyl ether; PBDE154: 2,2′,4,4′,5,6′-Hexabromodiphenyl ether; PBDE209: Decabromodiphenyl ether. ^a^ G-Mean (95%). ^b^ Median (25th, 75th percentiles).

## Data Availability

Data are publicly available on the NHANES website.

## Supplementary Materials

The following supporting information can be downloaded at: https://www.mdpi.com/article/10.3390/toxics12120852/s1, Figure S1: Pearson correlation coefficients among all variables; Figure S2: The continuous relationship of nine ln-transformed serum BFRs levels associated with LFTs in all participants based on RCS analysis. (A) AST; (B) ALB. Figure S3: The continuous relationship of nine ln-transformed serum BFRs levels associated with LFTs in all participants based on RCS analysis. (C) ALT; (D) ALP. Figure S4: The continuous relationship of nine ln-transformed serum BFRs levels associated with LFTs in all participants based on RCS analysis. (E) GGT; (F) TBIL. Figure S5: The continuous relationship of nine ln-transformed serum BFRs levels associated with LFTs in all participants based on RCS analysis. (G) TP; (H) SL. Figure S6: Association between ln-transformed serum BFRs mixture and the level of FIB-4, as assessed via quantile-based g-computation (first step) and weighted quantile sum regression (second step after exclusion of exposure factors with negative weights) (N = 4198). Table S1: Detection rates of serum BFRs. NHANES 2009–2014 (N = 4110). Table S2: Association between nine BFRs and biomarkers of liver injury in weighted linear regression (N = 4206). Table S3: Association between nine BFRs and biomarkers of liver injury in weighted linear regression (N = 4206). Table S4: Associations of BFRs index with three LFTs by WQS regression, NHANES (2009–2014). Table S5: Association between nine BFRs and biomarkers of liver injury in weighted linear regression (N = 4198).
