# Supplementary material for: The Association Between Brominated Flame Retardants Exposure and Liver-Related Biomarkers in US Adults
Source: Toxics. 2024 Nov 26;12(12):852. doi: 10.3390/toxics12120852 (PMC11679693; doi:10.3390/toxics12120852)
Supplement: Supplementary file 1 [file toxics-12-00852-s001.zip › toxics-3297038-supplementary.pdf]

Lists of Table

Table S1. Detection rates of serum BFRs. NHANES 2009-2014 (N = 4110).

Table S2. Association between nine BFRs and biomarkers of liver injury in weighted linear regression (N = 4206).

Table S3. Association between nine BFRs and biomarkers of liver injury in weighted linear regression (N = 4206).

Table S4. Associations of BFRs index with three LFTs by WQS regression, NHANES (2009-2014).

Table S5. Association between nine BFRs and biomarkers of liver injury in weighted linear regression (N = 4198).

Lists of Figure

Figure S1. Pearson correlation coefficients among all variables..

Figure S2. The continuous relationship of nine ln-transformed serum BFRs levels associated with LFTs in all participants based on RCS analysis. (A) AST; (B) ALB.

Figure S3. The continuous relationship of nine ln-transformed serum BFRs levels associated with LFTs in all participants based on RCS analysis. (C) ALT; (D) ALP.

Figure S4. The continuous relationship of nine ln-transformed serum BFRs levels associated with LFTs in all participants based on RCS analysis. (E) GGT; (F) TBIL.

Figure S5. The continuous relationship of nine ln-transformed serum BFRs levels associated with LFTs in all participants based on RCS analysis. (G) TP; (H) SL.

Figure S6. Association between ln-transformed serum BFRs mixture and the level of FIB-4, as assessed via quantile-based g-computation (first step) and weighted quantile sum regression (second step after exclusion of exposure factors with negative weights) (N = 4198).

Table S1. Detection rates of serum BFRs. NHANES 2009–2014 (N = 4110).

Serum BFRs (pg/g)	2009-2010			2011-2012			2013-2014		
	Pool number	< LLOD	Detection rate (%)	Pool number	< LLOD	Detection rate (%)	Pool number	< LLOD	Detection rate (%)
PBB153	301	39	87.04	251	15	94.02	284	22	92.25
PBDE28	301	7	97.67	251	11	95.62	284	0	100.00
PBDE47	301	0	100.00	251	0	100.00	284	0	100.00
PBDE85	301	34	88.70	251	29	88.45	284	35	87.68
PBDE99	301	0	100.00	251	0	100.00	284	00	100.00
PBDE100	301	0	100.00	251	0	100.00	284	0	100.00
PBDE153	301	0	100.00	251	0	100.00	284	0	100.00
PBDE154	301	61	79.73	251	25	90.04	284	37	86.97
PBDE209	301	12	96.01	251	4	98.41	284	6	97.89

LLOD: lower limit of detection.

Table S2. Association between nine BFRs and biomarkers of liver injury in weighted linear regression (N = 4206).

Group	The indicators of LFTs (liver function tests)											
	AST			ALB			ALT			ALP		
	β Coefficient (95% CI)			β Coefficient (95% CI)			β Coefficient (95% CI)			β Coefficient (95% CI)		
PBB153												
Q1(<1.891)	Reference			Reference			Reference			Reference		
Q2(≥1.891&<2.707)	**0.092**	**0.057**	**0.126**	0.006	-0.004	0.016	**0.139**	**0.089**	**0.189**	**0.035**	**0.003**	**0.066**
Q3(≥2.707&<3.301)	**0.098**	**0.046**	**0.149**	-0.005	-0.018	0.007	**0.122**	**0.058**	**0.186**	0.020	-0.023	0.063
Q4(≥3.301)	**0.080**	**0.032**	**0.128**	0.002	-0.012	0.017	**0.128**	**0.063**	**0.193**	0.011	-0.037	0.059
PBDE28												
Q1(<1.556)	Reference			Reference			Reference			Reference		
Q2(≥1.556&<1.944)	0.007	-0.029	0.042	-0.001	-0.009	0.007	-0.010	-0.056	0.036	-0.018	-0.047	0.012
Q3(≥1.944&<2.304)	0.023	-0.011	0.057	-0.001	-0.011	0.008	0.032	-0.008	0.072	-0.006	-0.039	0.028
Q4(≥2.304)	0.034	-0.010	0.078	-0.007	-0.017	0.003	0.026	-0.020	0.072	0.014	-0.019	0.047
PBDE47												
Q1(<4.404)	Reference			Reference			Reference			Reference		
Q2(≥4.404&<4.776)	0.012	-0.032	0.056	-0.007	-0.017	0.003	-0.003	-0.049	0.044	0.007	-0.025	0.039
Q3(≥4.776&<5.213)	0.023	-0.023	0.069	**-0.013**	**-0.021**	**-0.004**	0.031	-0.022	0.085	-0.005	-0.038	0.027
Q4(≥5.213)	0.013	-0.029	0.055	-0.009	-0.018	0.000	-0.004	-0.046	0.039	0.013	-0.022	0.047
PBDE85												
Q1(<0.395)	Reference			Reference			Reference			Reference		
Q2(≥0.395&<0.793)	-0.016	-0.059	0.026	-0.003	-0.011	0.005	-0.050	-0.105	0.004	-0.015	-0.051	0.021
Q3(≥0.793&<1.311)	0.029	-0.015	0.072	-0.007	-0.017	0.003	0.044	-0.007	0.096	0.003	-0.031	0.037
Q4(≥1.311)	-0.002	-0.048	0.044	-0.009	-0.018	0.000	-0.037	-0.084	0.011	0.003	-0.037	0.044
PBDE99												
Q1(<2.681)	Reference			Reference			Reference			Reference		

Q2(≥2.681&<3.104)	0.012	-0.034	0.059	-0.003	-0.013	0.007	0.018	-0.039	0.074	-0.003	-0.040	0.034
Q3(≥3.104&<3.619)	0.025	-0.016	0.066	-0.004	-0.015	0.006	**0.054**	**0.006**	**0.101**	-0.011	-0.046	0.025
Q4(≥3.619)	0.016	-0.030	0.062	**-0.012**	**-0.021**	**-0.004**	-0.001	-0.048	0.046	0.026	-0.011	0.063
PBDE100												
Q1(<2.776)	Reference			Reference			Reference			Reference		
Q2(≥2.776&<3.171)	0.029	-0.013	0.070	-0.009	-0.020	0.001	0.002	-0.042	0.046	-0.011	-0.046	0.023
Q3(≥3.171&<3.613)	0.025	-0.020	0.070	-0.005	-0.013	0.004	0.035	-0.011	0.081	0.005	-0.027	0.037
Q4(≥3.613)	0.014	-0.032	0.060	**-0.011**	**-0.021**	**-0.002**	-0.012	-0.054	0.030	0.001	-0.036	0.037
PBDE153												
Q1(<3.524)	Reference			Reference			Reference			Reference		
Q2(≥3.524&<3.932)	0.008	-0.041	0.056	0.008	-0.002	0.018	0.027	-0.034	0.088	0.011	-0.024	0.047
Q3(≥3.932&<4.415)	0.011	-0.036	0.057	0.007	-0.001	0.015	0.025	-0.033	0.083	0.006	-0.029	0.041
Q4(≥4.415)	0.030	-0.012	0.071	0.007	-0.001	0.016	0.044	-0.010	0.097	0.008	-0.028	0.044
PBDE154												
Q1(<0.349)	Reference			Reference			Reference			Reference		
Q2(≥0.349&<0.765)	0.040	-0.002	0.083	0.002	-0.007	0.010	0.031	-0.018	0.079	0.002	-0.035	0.040
Q3(≥0.765&<1.228)	0.030	-0.008	0.068	-0.005	-0.013	0.004	**0.050**	**0.001**	**0.099**	0.013	-0.019	0.045
Q4(≥1.228)	0.027	-0.019	0.072	**-0.010**	**-0.019**	**-0.001**	0.008	-0.044	0.060	0.014	-0.027	0.055
PBDE209												
Q1(<2.389)	Reference			Reference			Reference			Reference		
Q2(≥2.389&<2.721)	-0.021	-0.065	0.023	0.005	-0.003	0.013	-0.021	-0.079	0.036	-0.002	-0.037	0.034
Q3(≥2.721&<3.043)	0.041	-0.003	0.086	0.003	-0.006	0.011	**0.064**	**0.020**	**0.108**	0.008	-0.033	0.049
Q4(≥3.043)	0.028	-0.019	0.076	0.005	-0.004	0.015	0.049	-0.009	0.106	-0.017	-0.061	0.026

Table S3. Association between nine BFRs and biomarkers of liver injury in weighted linear regression (N = 4206).

Group	The indicators of LFTs (liver function tests)											
	GGT			TBIL			TP			SL		
	β Coefficient (95% CI)			β Coefficient (95% CI)			β Coefficient (95% CI)			β Coefficient (95% CI)		
PBB153												
Q1(<1.891)	Reference			Reference			Reference			Reference		
Q2(≥1.891&<2.707)	**0.198**	**0.116**	**0.281**	0.010	-0.032	0.051	-0.003	-0.012	0.006	**-0.047**	**-0.084**	**-0.010**
Q3(≥2.707&<3.301)	**0.275**	**0.188**	**0.361**	-0.025	-0.087	0.037	-0.010	-0.021	0.002	-0.024	-0.062	0.013
Q4(≥3.301)	**0.245**	**0.148**	**0.341**	-0.029	-0.101	0.042	-0.010	-0.021	0.001	**-0.048**	**-0.084**	**-0.012**
PBDE28												
Q1(<1.556)	Reference			Reference			Reference			Reference		
Q2(≥1.556&<1.944)	0.005	-0.081	0.092	0.010	-0.024	0.043	0.001	-0.007	0.009	0.017	-0.015	0.048
Q3(≥1.944&<2.304)	0.027	-0.039	0.093	0.015	-0.029	0.059	0.005	-0.002	0.013	-0.009	-0.039	0.020
Q4(≥2.304)	0.000	-0.082	0.082	0.019	-0.023	0.061	0.006	-0.004	0.017	0.008	-0.022	0.039
PBDE47												
Q1(<4.404)	Reference			Reference			Reference			Reference		
Q2(≥4.404&<4.776)	0.005	-0.063	0.073	-0.004	-0.036	0.028	0.001	-0.007	0.009	0.015	-0.012	0.042
Q3(≥4.776&<5.213)	0.013	-0.052	0.078	-0.006	-0.044	0.033	0.001	-0.008	0.010	-0.008	-0.039	0.022
Q4(≥5.213)	-0.022	-0.096	0.052	0.019	-0.020	0.057	0.005	-0.004	0.014	0.016	-0.016	0.049
PBDE85												
Q1(<0.395)	Reference			Reference			Reference			Reference		
Q2(≥0.395&<0.793)	-0.064	-0.134	0.007	-0.012	-0.055	0.032	0.004	-0.003	0.011	**0.034**	**0.006**	**0.061**
Q3(≥0.793&<1.311)	0.035	-0.021	0.092	-0.016	-0.053	0.020	0.005	-0.003	0.014	-0.016	-0.048	0.016
Q4(≥1.311)	-0.060	-0.142	0.022	-0.011	-0.052	0.030	0.005	-0.005	0.015	**0.034**	**0.006**	**0.063**
PBDE99												
Q1(<2.681)	Reference			Reference			Reference			Reference		

Q2(≥2.681&<3.104)	0.046	-0.029	0.121	-0.035	-0.075	0.005	0.004	-0.003	0.012	-0.005	-0.037	0.026
Q3(≥3.104&<3.619)	**0.066**	**0.021**	**0.110**	0.004	-0.034	0.042	0.004	-0.005	0.013	-0.028	-0.063	0.006
Q4(≥3.619)	0.015	-0.056	0.087	-0.016	-0.059	0.026	0.007	-0.003	0.018	0.016	-0.014	0.047
PBDE100												
Q1(<2.776)	Reference			Reference			Reference			Reference		
Q2(≥2.776&<3.171)	-0.011	-0.075	0.054	-0.014	-0.048	0.021	0.005	-0.003	0.012	0.027	0.000	0.053
Q3(≥3.171&<3.613)	0.027	-0.027	0.082	-0.027	-0.059	0.006	0.007	-0.002	0.015	-0.010	-0.038	0.018
Q4(≥3.613)	-0.045	-0.117	0.027	0.005	-0.032	0.042	0.006	-0.003	0.015	0.026	-0.005	0.057
PBDE153												
Q1(<3.524)	Reference			Reference			Reference			Reference		
Q2(≥3.524&<3.932)	-0.003	-0.086	0.079	0.021	-0.023	0.064	0.003	-0.004	0.011	-0.019	-0.050	0.012
Q3(≥3.932&<4.415)	0.025	-0.048	0.099	-0.034	-0.080	0.012	-0.002	-0.009	0.004	-0.014	-0.047	0.018
Q4(≥4.415)	0.035	-0.051	0.121	-0.010	-0.057	0.036	-0.004	-0.011	0.003	-0.014	-0.043	0.015
PBDE154												
Q1(<0.349)	Reference			Reference			Reference			Reference		
Q2(≥0.349&<0.765)	0.017	-0.057	0.091	0.017	-0.024	0.057	**0.009**	**0.002**	**0.015**	0.009	-0.017	0.036
Q3(≥0.765&<1.228)	**0.061**	**0.007**	**0.116**	0.002	-0.035	0.039	**0.010**	**0.003**	**0.017**	-0.020	-0.050	0.010
Q4(≥1.228)	-0.018	-0.098	0.061	0.009	-0.028	0.046	0.007	-0.002	0.017	0.019	-0.011	0.049
PBDE209												
Q1(<2.389)	Reference			Reference			Reference			Reference		
Q2(≥2.389&<2.721)	-0.020	-0.106	0.066	-0.032	-0.079	0.016	0.001	-0.007	0.009	0.000	-0.029	0.029
Q3(≥2.721&<3.043)	0.072	-0.006	0.151	0.015	-0.030	0.061	**0.009**	**0.002**	**0.017**	-0.023	-0.053	0.007
Q4(≥3.043)	0.052	-0.039	0.143	-0.007	-0.045	0.030	0.005	-0.002	0.012	-0.020	-0.052	0.012

Table S4. Associations of BFRs index with three LFTs by WQS regression, NHANES (2009-2014).

Outcomes	Direction	WQS model regression index weights									OR (95%CI)	P-value
AST	positive	0.215	0.2	0.197	0.17	0.113	0.073	0.013	0.011	0.008	1.015 (0.994,1.035)	0.157
	negative	0.331	0.296	0.177	0.139	0.042	0.010	0.001	0.001	0.001	1.002 (0.983,1.022)	0.813
	re-positive*	0.318	0.194	0.183	0.175	0.076	0.052				1.019 (0.998,1.040)	0.080
ALT	positive	0.397	0.245	0.154	0.143	0.029	0.016	0.014	0.001	0.001	**1.049 (1.020,1.078)**	**0.001**
	negative	0.436	0.314	0.127	0.041	0.034	0.025	0.012	0.011	0.001	0.998 (0.979,1.018)	0.874
	re-positive*	0.419	0.241	0.150	0.144	0.029	0.018				**1.050 (1.021,1.079)**	**0.001**
GGT	positive	0.431	0.348	0.139	0.033	0.025	0.022	0.002	0.000	0.000	**1.055 (1.013,1.099)**	**0.011**
	negative	0.396	0.201	0.181	0.158	0.049	0.010	0.003	0.002	0.000	1.010 (0.984,1.036)	0.474
	re-positive*	0.436	0.351	0.135	0.032	0.025	0.022				**1.055 (1.013,1.099)**	**0.010**

Notes: WQS: weighted quantile sum; OR: odds ratio; CI: confidence interval. All of the models are adjusted for demographic characteristics (gender, age, race, educational levels, marital status and PIR), lifestyle (BMI categories, cotinine levels and alcohol consumption) and self-reported of hypertension, diabetes conditions and activity. *re-positive: the model in WQS regression was obtained by excluding pollutants with negative direction as indicated in quantile-based g computation (QGC).

Table S5. Association between nine BFRs and biomarkers of liver injury in weighted linear regression (N = 4198).

Group	FIB-4			
	β Coefficient (95%CI)			P
PBB153				
Q1(<1.891)	Reference			
Q2(≥1.891&<2.707)	0.004	-0.010	0.018	0.575
Q3(≥2.707&<3.301)	**0.020**	**0.004**	**0.036**	**0.014**
Q4(≥3.301)	0.016	-0.005	0.038	0.123
PBDE28				
Q1(<1.556)	Reference			
Q2(≥1.556&<1.944)	0.009	-0.003	0.020	0.130
Q3(≥1.944&<2.304)	0.008	-0.007	0.023	0.275
Q4(≥2.304)	0.006	-0.014	0.026	0.568
PBDE47				
Q1(<4.404)	Reference			
Q2(≥4.404&<4.776)	**0.017**	**0.002**	**0.031**	**0.024**
Q3(≥4.776&<5.213)	**0.013**	**0.0002**	**0.025**	**0.046**
Q4(≥5.213)	0.006	-0.011	0.023	0.455
PBDE85				
Q1(<0.395)	Reference			
Q2(≥0.395&<0.793)	-0.007	-0.023	0.010	0.407
Q3(≥0.793&<1.311)	0.004	-0.015	0.023	0.653
Q4(≥1.311)	-0.006	-0.024	0.013	0.554
PBDE99				
Q1(<2.681)	Reference			
Q2(≥2.681&<3.104)	-0.0004	-0.017	0.016	0.957
Q3(≥3.104&<3.619)	-0.0008	-0.015	0.014	0.910
Q4(≥3.619)	-0.0050	-0.024	0.015	0.629
PBDE100				
Q1(<2.776)	Reference			

Q2(≥2.776&<3.171)	0.008	-0.005	0.021	0.227
Q3(≥3.171&<3.613)	0.007	-0.006	0.019	0.300
Q4(≥3.613)	-0.003	-0.020	0.014	0.732
PBDE153				
Q1(<3.524)	Reference			
Q2(≥3.524&<3.932)	-0.018	-0.036	-0.0009	0.040
Q3(≥3.932&<4.415)	-0.003	-0.019	0.013	0.712
Q4(≥4.415)	-0.011	-0.030	0.008	0.244
PBDE154				
Q1(<0.349)	Reference			
Q2(≥0.349&<0.765)	0.008	-0.008	0.023	0.329
Q3(≥0.765&<1.228)	0.014	-0.003	0.030	0.104
Q4(≥1.228)	0.002	-0.018	0.021	0.870
PBDE209				
Q1(<2.389)	Reference			
Q2(≥2.389&<2.721)	-0.024	-0.038	-0.010	0.001
Q3(≥2.721&<3.043)	-0.032	-0.051	-0.013	0.002
Q4(≥3.043)	-0.021	-0.040	-0.003	0.027

Figure S1

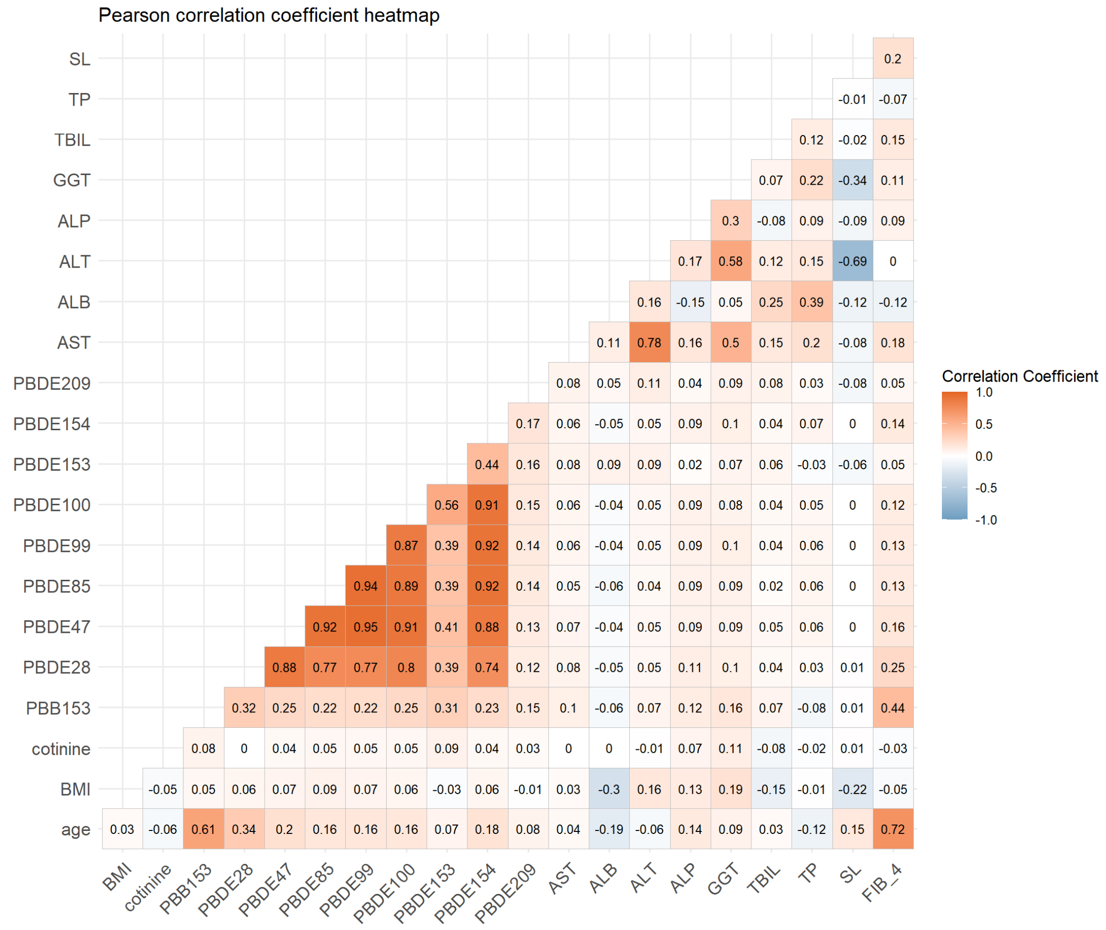

Figure S1. Pearson correlation coefficients among all variables.

Figure S2

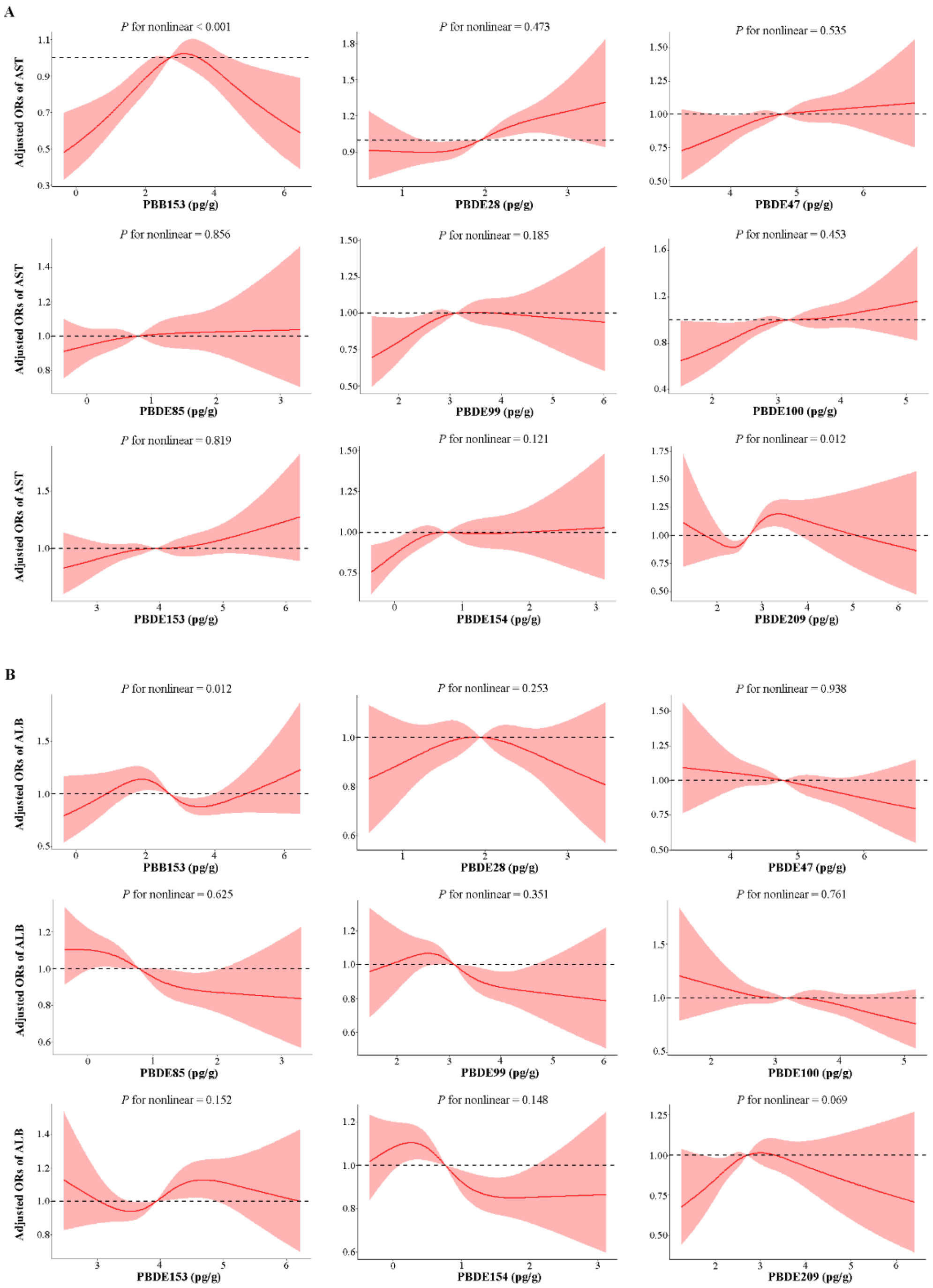

Figure S2. The continuous relationship of nine ln-transformed serum BFRs levels associated with LFTs in all participants based on RCS analysis. (A) AST; (B) ALB.

Figure S3

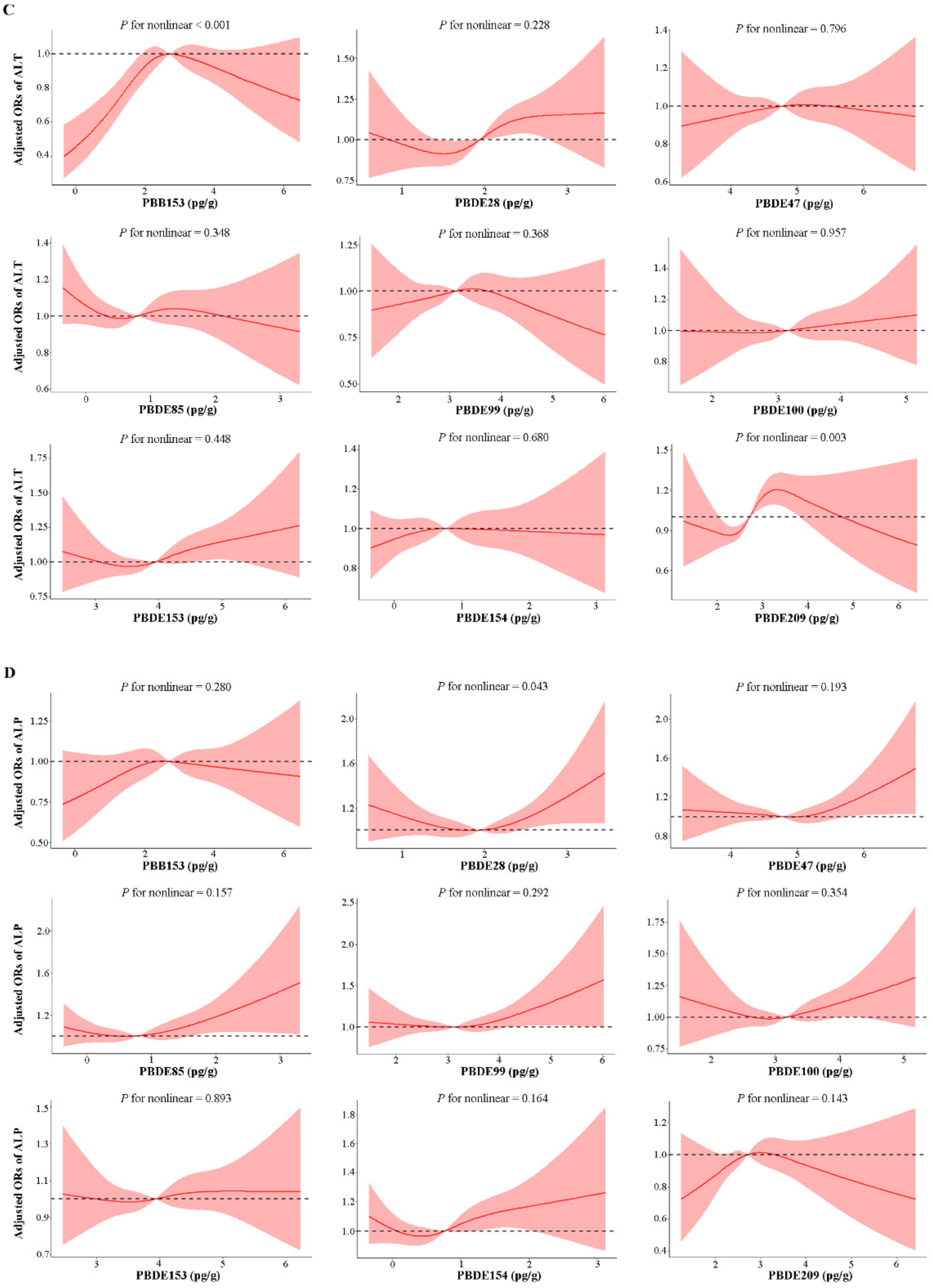

Figure S3. The continuous relationship of nine ln-transformed serum BFRs levels associated with LFTs in all participants based on RCS analysis. (C) ALT; (D) ALP.

Figure S4

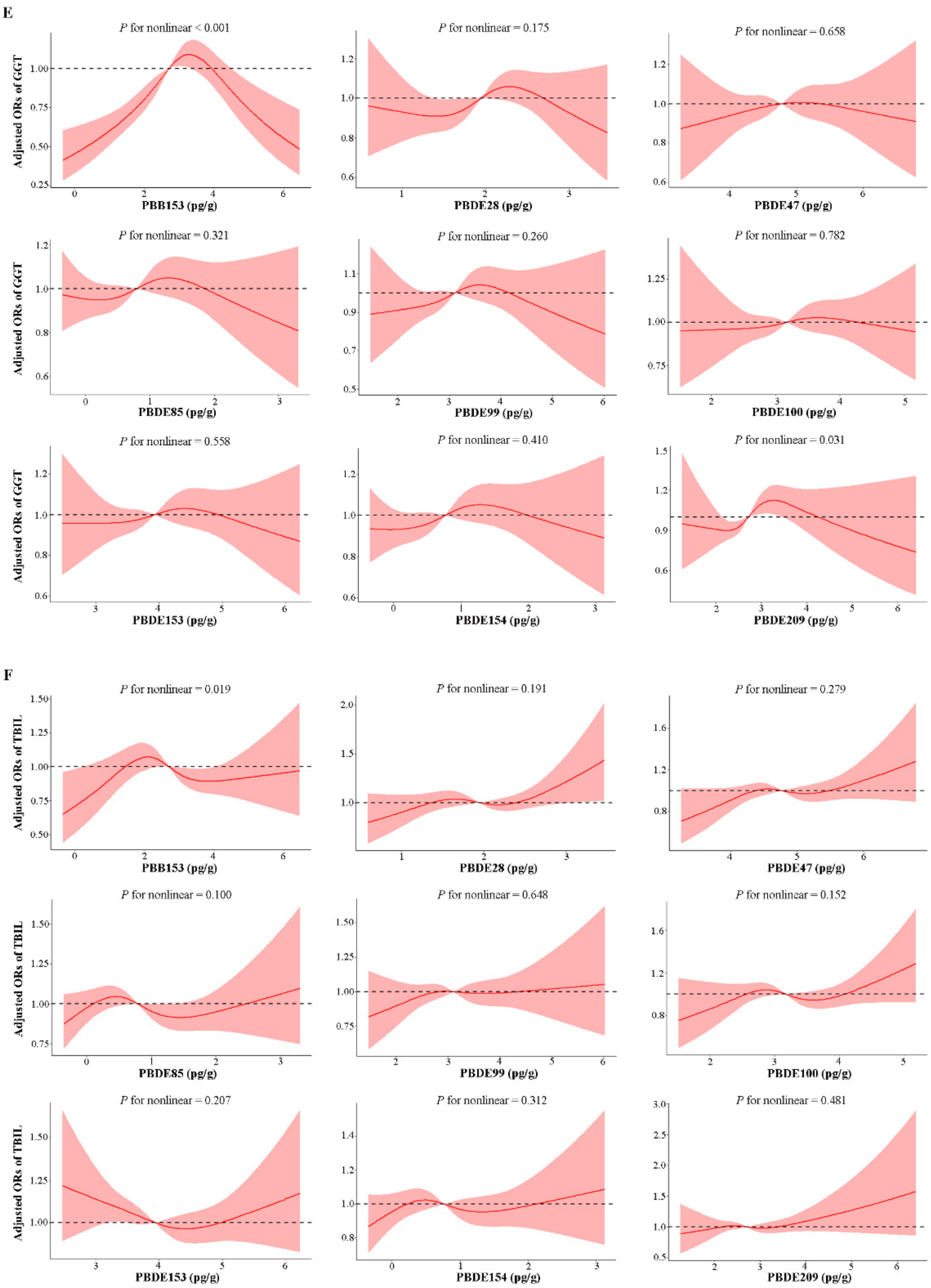

Figure S4. The continuous relationship of nine ln-transformed serum BFRs levels associated with LFTs in all participants based on RCS analysis. (E) GGT; (F) TBIL.

Figure S5

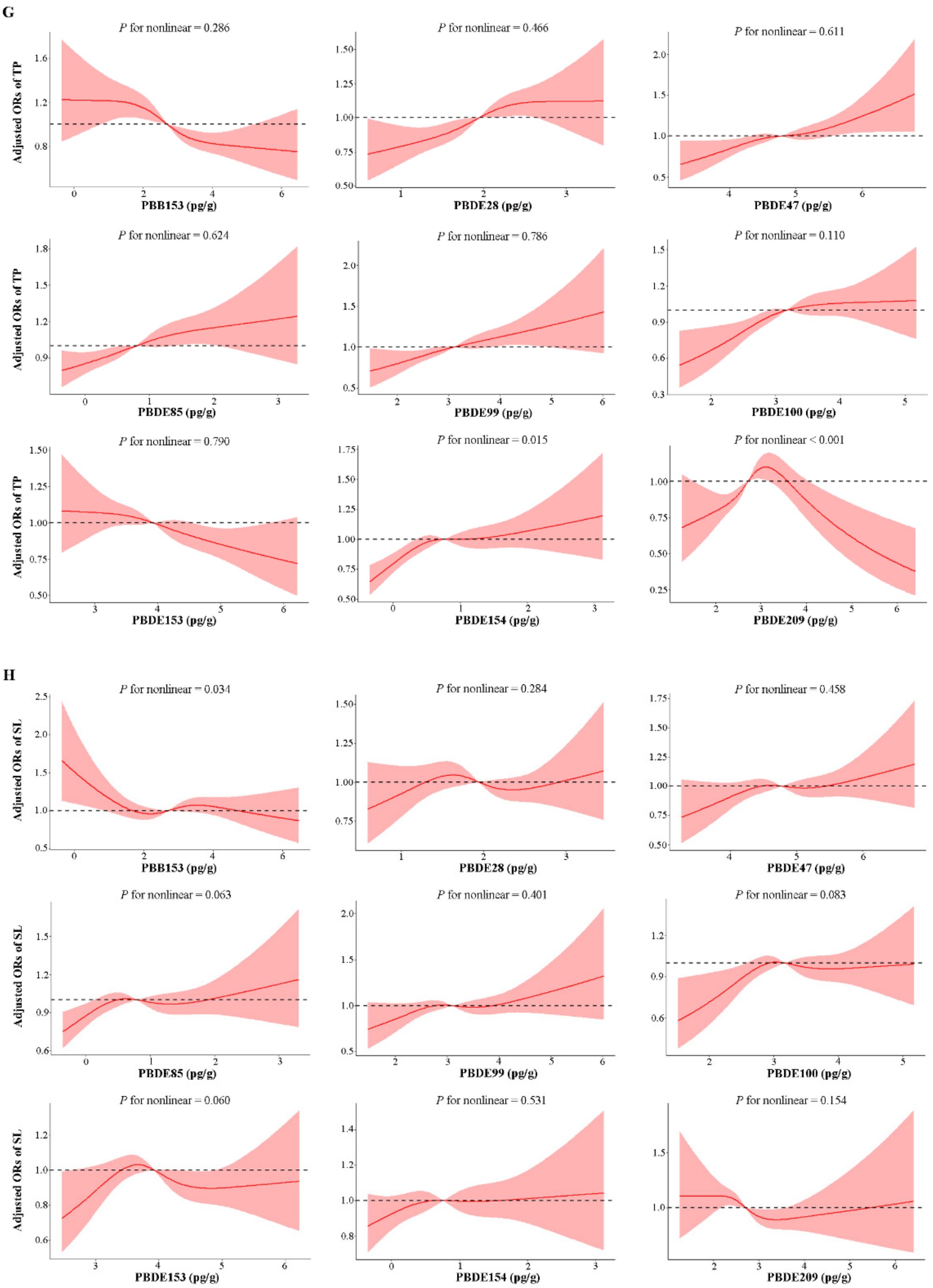

Figure S5. The continuous relationship of nine ln-transformed serum BFRs levels associated with LFTs in all participants based on RCS analysis. (G) TP; (H) SL.

Figure S6

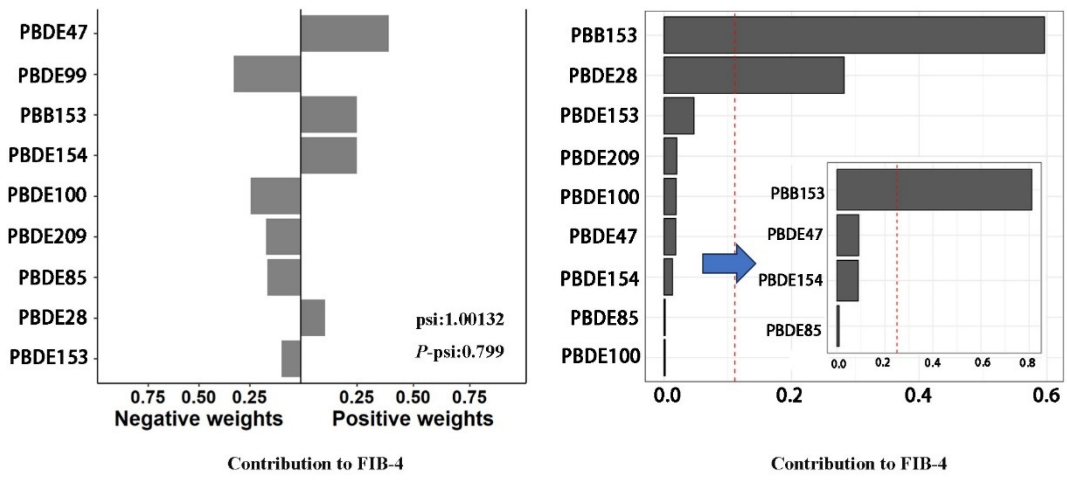

Figure S6. Association between ln-transformed serum BFRs mixture and the level of FIB-4, as assessed via quantile-based g-computation (first step) and weighted quantile sum regression (second step after exclusion of exposure factors with negative weights).
